# Characterization of a Capsule-Deficient *Pasteurella multocida* Isolated from *Cygnus melancoryphus*: Genomic, Phenotypic, and Virulence Insights

**DOI:** 10.3390/microorganisms13051024

**Published:** 2025-04-29

**Authors:** Nansong Jiang, Hongmei Chen, Weiwei Wang, Qizhang Liang, Qiuling Fu, Rongchang Liu, Guanghua Fu, Chunhe Wan, Yu Huang, Longfei Cheng

**Affiliations:** 1 Research Center for Poultry Diseases, Institute of Animal Husbandry and Veterinary Medicine, Fujian Academy of Agricultural Sciences, Fuzhou 350013, China; nansongjiang@126.com (N.J.); chenhmei052@126.com (H.C.); wangweiweihn@163.com (W.W.); 11817043@zju.edu.cn (Q.L.); qiulingfu0822@163.com (Q.F.); liurongc@foxmail.com (R.L.); fuyuan163@163.com (G.F.); chunhewan@126.com (C.W.); huangyu_815@163.com (Y.H.); 2 Fujian Key Laboratory for Prevention and Control of Avian Diseases, Fuzhou 350013, China; 3 Fujian Industry Technology Innovation Research Academy of Livestock and Poultry Diseases Prevention & Control, Fuzhou 350013, China

**Keywords:** *Pasteurella multocida*, *Cygnus melancoryphus*, capsule deficiency, FCF147 strain, adaptation, virulence

## Abstract

*Pasteurella multocida* is a zoonotic pathogen responsible for severe diseases in domestic and wild animals, posing threats to public health and causing substantial economic losses. Here, we describe a naturally attenuated *P. multocida* strain, FCF147, isolated from a mortality event involving black-necked swans (*Cygnus melancoryphus*) in a wildlife habitat in Fujian, China. Genomic and phylogenetic analyses revealed that FCF147 is evolutionarily distant from other *P. multocida* lineages and lacks the entire capsule gene cluster. Morphological observations revealed that the loss of the capsule exposed proteins on the bacterial surface. Phenotypic characterization demonstrated reduced capsule production, enhanced biofilm formation, and increased tolerance to heat stress. In vivo infection models confirmed that FCF147 exhibits markedly attenuated virulence in both mice and poultry. However, immunization with FCF147 did not provide effective protection against the challenge of a virulent capsular type A strain. These findings suggest that while FCF147 is poorly virulent, its ability to form robust biofilms and survive thermal stress may facilitate persistence in wild bird reservoirs and potential transmission routes. These findings offer novel insights into the ecological adaptation and pathogenic potential of naturally capsule-deficient *P. multocida* in wildlife, highlighting their relevance to wildlife surveillance and disease ecology.

## 1. Introduction

*Pasteurella multocida* is a notorious encapsulated, Gram-negative coccobacillus with a broad host range, encompassing human, domestic, and wild animals [1]. Clinical manifestations associated with *P. multocida* infection can be broadly categorized into two types: (i) respiratory disorders, such as atrophic rhinitis in pigs and rabbits, and (ii) systemic infections, including fowl cholera in poultry and sepsis in humans through wound contamination [1,2,3,4]. Numerous studies have documented that *P. multocida* has caused significant mortality events in wild animals, including Saiga Antelope (*Saiga tatarica*) [5], African elephants (*Loxodonta africana*) [6], albatross (*Thalassarche carteri*), and penguins (*Eudyptes moseleyi*) [7]. Importantly, wild birds play a critical role in pathogen dissemination, posing risks to agriculture and public health [8,9,10]. Although *P. multocida* is not considered part of the normal microbiota of birds, it can also act as a highly invasive pathogen [11]. Birds that recover from infection and develop specific immunity may remain colonized by *P. multocida* or directly carry low-virulence strains, both of which enable asymptomatic carriers to transmit the bacterium to nonimmune birds and other animals [12,13,14,15,16]. Given the increasing concerns about zoonotic transmission and pathogen evolution in wildlife [8,9,10], continuous surveillance of *P. multocida* dynamics in wild populations is essential for assessing regional epidemic risks.

The pathogenicity, host preference, and prevalence of *P. multocida* are primarily associated with its capsular serogroups (A, B, D, E, and F), lipopolysaccharide (LPS) types (1 to 16), and the multi-locus sequence typing (MLST) [1,17]. *P. multocdida* with capsular serogroups A, D, and F are commonly linked to fowl cholera, conjunctivitis, and respiratory disorders such as rhinitis, pneumonia, and shipping fever, while strains with genotypes B and E are more frequently associated with bovine hemorrhagic septicemia [4]. Our previous study has shown that the combined typing A: L1: ST129 of *P. multocida* is the dominant clone in Chinese poultry [18]. The capsular polysaccharide (CPS), a crucial virulence factor, plays a pivotal role in the ecological dominance and epidemiological success of *P. multocida* by concealing surface antigens from the host immune system, inhibiting phagocytosis, and enhancing resistance to bactericidal activity [1,4,17]. Empirical studies have demonstrated that CPS is indispensable for full virulence in both poultry and mice [19,20], and genetic modifications of the capsule gene loci have been investigated as major strategies for developing attenuated vaccine strains [19,20,21]. Nevertheless, some *P. multocida* strains remain untypable for capsular classification while retaining their prevalence and virulence [4,22,23], suggesting alternative mechanisms of adaptation. For example, researchers identified three *P. multocida* strains that had naturally lost their capsule synthesis ability due to *fis* gene mutations [24]. However, to date, reports on naturally capsule-deficient *P. multocida* strains remain scarce, and their epidemiology, environmental adaptability, and virulence remain largely unexplored.

We isolated a capsule-deficient *P. multocida* strain from a mortality event in a black-necked swan (*Cygnus melancoryphus*) population at a wildlife habitat in Fujian Province, China. Utilizing bioinformatics analysis, phenotype determination, pathogenicity assessment, and immune protection assay, we characterized its phylogenetic relationships, environmental adaptability, and virulence. Overall, our findings reveal that the swan-derived *P. multocida* strain (i) lacks the entire capsule gene locus and is phylogenetically distant from *P. multocida* genomes of various types, (ii) demonstrates enhanced biofilm formation and increased tolerance to heat stress, and (iii) exhibits significantly reduced virulence in poultry and mice compared to capsular type A strains, but fails to provide effective protection against challenge with a capsular type A strain. These results provide novel insights into the ecological adaptation and pathogenic potential of naturally capsule-deficient *P. multocida* in wildlife populations.

## 2. Materials and Methods

### 2.1. Bacterial Strains, Culture Conditions, and Reagents

*P. multocida* strains used in this study for comparison included FCF12 (D: L6: ST50), FCF15 (A:L1:ST129), FCF79 (F:L3:ST176), FCF147 (-:L2:-), which were isolated from pig, duck, monkey, and wild swan (*Cygnus melancoryphus*), respectively. Unless specified otherwise, *P. multocida* strains were cultured on tryptic soy agar (TSA; BD Difco^TM^, Franklin Lakes, NJ, USA) or in tryptic soy broth (TSB; BD Difco^TM^, Franklin Lakes, NJ, USA) supplemented with 5% fetal bovine serum (FBS; Gibco, ThermoFisher, Waltham, MA, USA) at 37 °C for a minimum of 12 h. Unless otherwise specified, all chemical reagents used in this study were purchased from Macklin Biochemical Technology Co., Ltd. (Shanghai, China).

### 2.2. Whole Genome Sequencing (WGS), Genotype Determination, and Phylogeny

DNA extraction, library construction, quality control, data polishing, assembly, and SNP calling were all conducted as outlined in our previous study [18]. Paired-end reads of FCF147 were generated on a PacBio RSII sequencer (Sinobiocore Biotechnology Co., Ltd., Beijing, China). Comprehensive information on all genomes used in this study is listed in Table S1. The biological functions of genes were annotated employing the Gene Ontology (GO) [25] and the Kyoto Encyclopedia of Genes and Genomes (KEGG) [26] databases. Resistance genes, virulence genes, and plasmid replication sites were identified using ABRicate v1.0.1.30 (https://github.com/tseemann/abricate, accessed on 26 April 2025) with a 90% coverage threshold against the Comprehensive Antibiotic Resistance Database (CARD) [27], the Virulence Factor Database (VFDB) [28], the Pathogen Host Interactions (PHI) [29], and PlasmidFinder [30], respectively. Blastn [31] was employed to determine the capsular and LPS genotypes with a 90% coverage cut-off.

Publicly available WGS data of an additional 297 *P. multocida* isolates for comparative analysis were retrieved from the Sequence Read Archive (n = 206), RefSeq (n = 89) databases, which were isolates from the USA (n = 129), Australia (n = 39), and Bangladesh (n = 3), with the remainder from China. Our selection focused on the geographic, host, and sequence-type diversity to ensure a comprehensive representation of *P. multocida* population structure. This dataset size was sufficient to enable robust phylogenetic inference and genomic comparison, as supported by our previous study [18]. The genomic information, dataset composition, and sources of all isolates included in this study are detailed in Table S1. A maximum likelihood (ML) phylogeny was inferred from the alignment of core SNPs (n = 2,682,091) across the 298 genomes using RAxML v8.1.23 [32] with a general time-reversible (GTR) model, as previously described [33].

### 2.3. Transmission Electron Microscope (TEM) and Scanning Electron Microscope (SEM) Analysis

TEM and SEM were performed to examine the morphological characteristics of *P. multocida*. Bacterial cultures were harvested at the mid-log phase and washed twice with phosphate-buffered saline (PBS). For TEM analysis, the samples were initially fixed in 2.5% glutaraldehyde at 4 °C overnight, followed by post-fixation with 1% osmium tetroxide for 1 h. The samples were then dehydrated through a graded ethanol series ranging from 30% to 100% and embedded in epoxy resin. Ultrathin sections, measuring 70–90 nm, were prepared using an ultramicrotome, stained with 2% uranyl acetate and lead citrate, and observed with a TEM (H-7650, HITACHI, Tokyo, Japan) at an accelerating voltage of 80 kV. For SEM analysis, bacterial cells were fixed in 2.5% glutaraldehyde at 4 °C for a duration of 12 h, followed by post-fixation with 1% osmium tetroxide for 1 h. The samples were dehydrated using a graded ethanol series and were subjected to critical point drying with CO_2_. After mounting on aluminum stubs, the samples were sputter-coated with gold and visualized using an SEM (SU8100, HITACHI, Tokyo, Japan) at an accelerating voltage of 3.0 kV.

### 2.4. One-Step In Vitro Growth Curve

Bacterial proliferation was assessed by monitoring the optical density (OD) at regular intervals. Cultures of *P. multocida* in the mid-log phase were diluted 1:100 to achieve an OD600 of 0.1 in TSB and incubated at 37 °C with shaking at 200 revolutions per minute (rpm). OD600 measurements were recorded every 30 min over a 24-h period using a microplate reader (Tecan Infinite^®^ 200 PRO, Tecan Group Ltd., Männedorf, Switzerland). Growth curves were generated to facilitate the comparison of proliferation rates among different strains.

### 2.5. Capsule and Biofilm Quantification

A modified quantification assay was employed to evaluate capsule production in *P. multocida* strains, as previously described [23]. CPS extraction was conducted by resuspending mid-exponential phase cultures in 800 µL of PBS and 200 µL of capsule extraction buffer (500 mM citric acid, pH 2.0, 1% wt/vol Zwittergent^®^ 3-10). Bacterial enumeration was performed through serial tenfold dilutions and colony-forming unit (CFU) counts. The bacterial suspension was incubated at 50 °C for 30 min, followed by centrifugation at 13,000× *g* for 10 min to collect the supernatant. A 300 µL aliquot of the supernatant was mixed with 1 mL of absolute ethanol and incubated at 4 °C for 30 min to precipitate the capsular material. The precipitate was collected by centrifugation at 13,000× *g*, dried, and resuspended in 300 µL of double-distilled water. Capsular polysaccharides were quantified using a stain-all assay. Specifically, each 100 µL sample was mixed with 900 µL of staining solution (0.2 mg/mL stains-all, 0.06% glacial acetic acid, 50% formamide) and immediately measured at 640 nm. A standard curve, ranging from 0.5 to 5 µg per 100 µL, was prepared under identical conditions. Capsule concentrations were ascertained by comparing the absorbance values to the standard curve.

A modified Crystal Violet (CV) staining method was utilized to quantify biofilm biomass as previously described [34]. Briefly, *P. multocida* strains grown to the mid-log phase were collected by centrifugation at 6000× *g* for 10 min and subsequently adjusted to an OD600 of 0.5. Following this, 800 µL of each culture was transferred in triplicate into 48-well cell culture plates and incubated under aerobic and static conditions for 48 h at 37 °C. Post incubation, the cultures in each well were carefully removed, and the wells were gently washed three times with PBS. An amount of 800 μL of methanol was added to fix the adhered bacterial cells. The content of the wells was removed after 30 min, and the plates were dried at room temperature. Next, 800 μL of 1% crystal violet dye was added to each well and incubated for 30 min, followed by washing the plates three times in PBS and air drying. The biofilm formed in the plates was quantified by recording the absorbance at 595 nm after the addition of 800 μL of 33% glacial acetic acid for 15 min at room temperature.

### 2.6. Stress Tolerance Testing Assay

To evaluate the tolerance of *P. multocida* to oxidative, thermal, acidic/alkaline, and osmotic stress, cultures in the mid-log growth phase were adjusted to an OD600 of 0.5 and subsequently exposed to varying conditions: 0–40 mM hydrogen peroxide (H_2_O_2_), temperature ranging from 37 to 60 °C, pH levels from 2 to 12, and sodium chloride concentrations from 0 to 2M NaCl. These cultures were incubated for one hour under each condition, respectively. Following incubation, serial dilutions were plated on TSA, and CFUs were enumerated after 24 h of incubation. For the assessment of desiccation tolerance, 100 µL of bacterial suspension was spotted onto sterile autoclaved filter paper placed in a Petri dish containing color-changing silica gel (Solarbio Science & Technology Co., Ltd., Beijing, China) as the desiccant. The plates were sealed with parafilm and incubated at 37 °C for durations of 1, 2, 3, and 4 h. Post-incubation, the desiccated bacterial samples were resuspended in 1 mL of PBS, followed by serial dilutions plating on TSA and CFU enumeration after a 24-h incubation period.

### 2.7. Determination of Minimum Inhibitory Concentration (MIC)

To assess the antimicrobial susceptibility of *P. multocida*, the broth microdilution method was performed according to the CLSI M100-Ed35 guidelines [35]. Briefly, 14 commonly used veterinary antibiotics were serially diluted in cation-adjusted Mueller–Hinton broth (CA-MHB; BD Difco^TM^, Franklin Lakes, NJ, USA) and mixed with an equal volume of bacterial suspension (~1.5 × 10⁶ CFU/mL) in a sterile, clear 96-well microtiter plate. *Escherichia coli* (ATCC^®^ 25922^TM^) served as the quality control strain. Following an 18-h incubation period at 37 °C, the MIC values were determined as the lowest antibiotic concentrations that completely inhibited visible bacterial growth.

### 2.8. Animal Pathogenicity and Immune Protection Assay

The animal experimental procedure was approved by the Research Ethics Committee of the Institute of Animal Husbandry and Veterinary Science of the Fujian Academy of Agricultural Sciences (Approval No. MYLISC2024-019, Approval Date 19 December 2024). The number of animals used in each group was determined based on ethical considerations and the expected magnitude of phenotypic differences in survival and virulence among strains. These sample sizes were sufficient to detect consistent and reproducible differences in pathogenicity and immune protection between FCF147 and other strains while minimizing unnecessary animal use in accordance with the 3Rs principle. All animals were confirmed to be clinically healthy and specific-pathogen-free before these experiments. Mice and chicken/ducks were respectively housed in standard plastic cages with wood shavings and well-ventilated poultry houses at a controlled temperature of 22 ± 2 °C, relative humidity of 50–60%, and a 12 h light/dark cycle. All animals were provided with commercial pelleted feed and water ad libitum. Animals were monitored twice daily. Humane endpoints included severe clinical signs, triggering euthanasia via chloral hydrate injection. Analgesics/anesthetics were withheld to prevent immune response interference. The investigators were not blinded to group allocation during the experiments or outcome assessment. All outcome measurements were based on objective criteria (e.g., survival rates, quantitative assays), reducing the risk of observer bias.

To evaluate the pathogenicity of FCF147, infection experiments were performed on mice and three commonly reared poultry species in China. For the mouse pathogenicity assay, ICR mice (male, 6–8 weeks old, weighing 25–30 g, n = 120) were purchased from the Laboratory Animal Center, Fujian Agriculture and Forestry University, Fujian, China. All mice were randomly divided into six groups (n = 20 per group) and intraperitoneally injected with 100 µL of bacterial suspension containing either FCF12 (96 CFU), FCF15 (84 CFU), FCF79 (65 CFU), FCF147 (67 CFU), and FCF147 (~10^9^ CFU), with PBS group (n = 20) serving as control. For the poultry pathogenicity assay, 90-day-old male yellow-feather broilers (n = 30), shelducks (n = 30), and Cherry Valley ducks (n = 30) sourced from local commercial farms were used. Each species was randomly divided into two groups (n = 10 per group) and intramuscularly injected with 100 µL of bacterial suspension containing either FCF15 (84 CFU) or FCF147 (~10^9^ CFU), with the PBS group (n = 10) serving as a control.

All animals that survived the infection procedure were included in subsequent immunization and challenge experiments. No animals were excluded during the course of this study. Surviving mice (n = 12) from the FCF147 (~10^9^ CFU) group were selected and maintained for an additional 11 days prior to undergoing an immune protection assay. These mice were randomly divided into three groups (n = 4 per group), with age-matched healthy mice serving as controls. Each group was then intraperitoneally injected with 100 µL of the following bacterial suspension: FCF12 (83 CFU), FCF15 (66 CFU), and FCF79 (72 CFU). Similarly, the surviving chickens (n = 10) and ducks (n = 10 in each group) from the FCF147 (~10^9^ CFU) group were kept for an additional 11 days before being intramuscularly injected with 100 µL of FCF15 (75 CFU) bacterial suspension to assess survival, with age-matched poultry as controls.

### 2.9. Histopathological Examination

To assess histopathological changes, the hearts, livers, lungs, and kidneys were collected from surviving or deceased ducks within each group. These organs were fixed in 4% paraformaldehyde, dehydrated, embedded in paraffin, sectioned into 4 μm-thick sections, placed on microscope slides, and stained with hematoxylin and eosin (HE) using standard procedures. Light microscopy was employed to assess the severity of pathological changes, focusing on tissue architecture, congestion, inflammatory infiltration, hemorrhage, degeneration, and necrosis.

### 2.10. Statistical Analyses

The data are presented as means ± standard deviations (SDs) and were subjected to analysis using one-way ANOVA followed by Dunnett’s multiple comparison test in GraphPad Prism v10.3.1 (GraphPad Software Inc., San Diego, CA, USA). Survival curves were analyzed using Kaplan–Meier analysis in GraphPad Prism. Statistical significance was determined at a *p*-value of <0.05 (*), <0.01 (**), <0.001 (***), or <0.0001 (****), while *p*-values exceeding 0.05 were regarded as not significant (ns).

## 3. Results

### 3.1. Genomic and Phylogenetic Characterization of FCF147

A mortality event involving multiple black-necked swans occurred at a wildlife habitat in Fujian, China (Figure S1a). Post-mortem examinations revealed pathological lesions consistent with fowl cholera, characterized by severe pericardial hemorrhage (Figure S1b), intestinal swelling and hemorrhagic exudates with a gelatinous appearance (Figure S1c), and hepatomegaly with extensive necrosis (Figure S1d). A *P. multocida* strain, FCF147, was isolated and identified from these lesions.

WGS analysis revealed that FCF147 harbors a 34,205 bp plasmid (Figure S2). Comparative genomic analysis classified FCF147 as an L2-type LPS strain; however, it could not be assigned to any known capsular serogroup or MLST category. Gene annotations from the GO and KEGG databases indicated that genes related to “Cell motility” were present, suggesting potential flagellum or adhesion-associated factors that might contribute to host invasion (Figure S3a). The enrichment of metabolic pathways and the biosynthesis of secondary metabolites imply that FCF147 possesses significant adaptive capabilities and may regulate virulence through metabolic control mechanisms (Figure S3b,c). A substantial proportion of genes in the COG annotation were categorized as “Function unknown” or “General function prediction only”, suggesting that FCF147 may contain numerous uncharacterized genes or potential virulence factors that warrant further investigation (Figure S3d). Among the virulence-associated genes, those related to endotoxins and immune evasion were predominant, suggesting a certain degree of pathogenic potential (Figure S3e). PHI annotation further revealed that FCF147 shares strong protein homology with *Salmonella enterica* and *Escherichia coli* (Figure S3f). However, genome screening did not identify any known antimicrobial resistance gene in FCF147 (Table S1).

**Figure 1:**
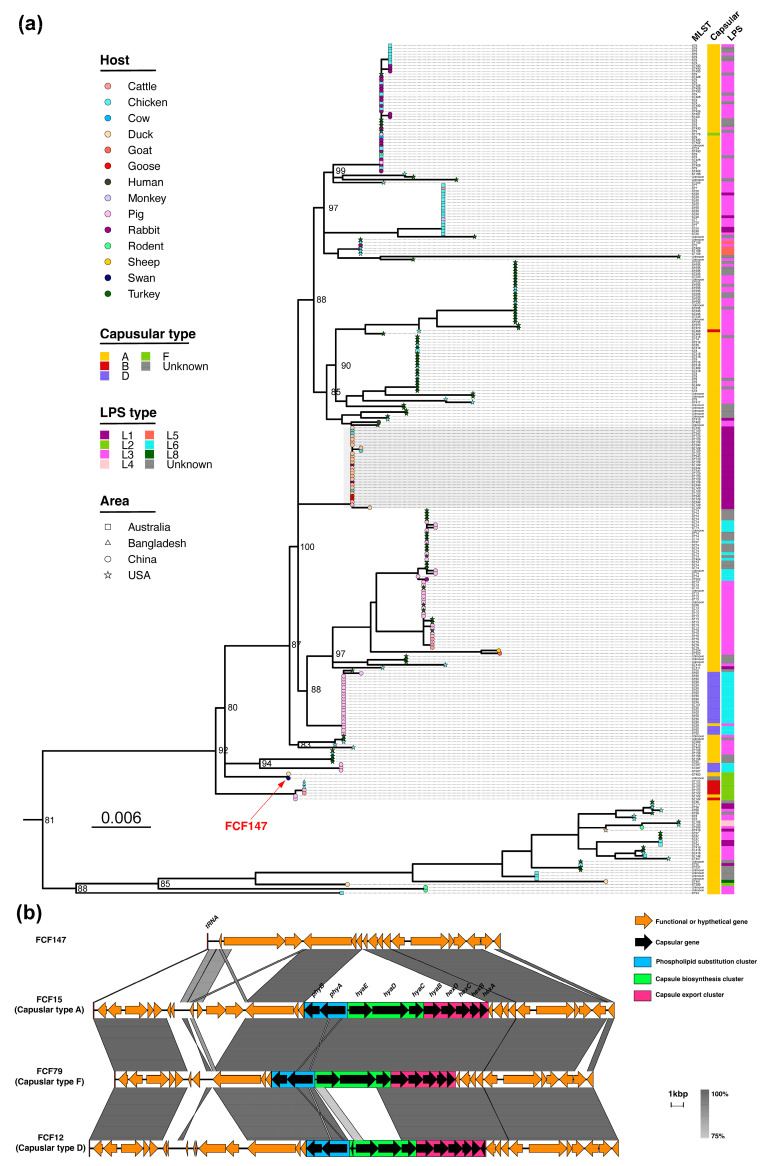
Comparative analysis of FCF147 genome. (**a**) A midpoint-rooted ML phylogenetic tree of *P. multocida* was constructed using the alignment of core SNPs (n = 2,682,091) across 298 genomes. The branch tips are shaped according to geographic regions and colored based on the host species of isolates. The aligned annotations correspond to capsular genotypes, LPS genotypes, and MLST profiles, respectively. The gray shaded area in the figure represents the clade corresponding to the predominant A: L1: ST129 clone cluster. (**b**) The sequence comparison of four *P. multocida* capsule gene loci is annotated and aligned. Regions with 75–100% nucleotide sequence identity are indicated by shading. ORFs are depicted as arrows, indicating the direction of transcription. Different colors represent distinct genes, and the colored regions denote different capsule gene modules.

To determine the phylogenetic position of FCF147 within the *P. multocida* population, a midpoint-rooted phylogenetic tree was constructed using core SNPs from 298 *P. multocida* genomes, encompassing four capsular types, seven LPS types, and 64 MLST types. The ML tree was well supported, with bootstrap values exceeding 80% for both major and minor clades (Figure 1a). FCF147 was positioned adjacent to a Chinese duck origin strain classified as A:L2:ST400, yet did not cluster closely with the *P. multocida* strain of a different source or serotype. Notably, FCF147 was phylogenetically distant from the predominant A: L1: ST129 clone identified in Chinese poultry. Based on clinical findings and WGS analysis, FCF147 appears to be a novel, non-epidemic *P. multocida* strain with potential pathogenicity.

### 3.2. Loss of Capsule Locus and Associated Morphological Changes

To explore the relationship between capsule genes and virulence in FCF147, we analyzed its capsule gene locus (Figure 1b). Unexpectedly, our findings revealed that FCF147 inherently lacks the entire capsule gene cluster, encompassing the phospholipid substitution cluster, capsule biosynthesis cluster, and capsule export cluster. The genomic regions flanking the absent capsule locus exhibit a high degree of similarity to those found in *P. multocida* strains with capsular types A, F, and D. This genetic deletion elucidates why FCF147 cannot be classified into any recognized capsule serotype.

**Figure 2:**
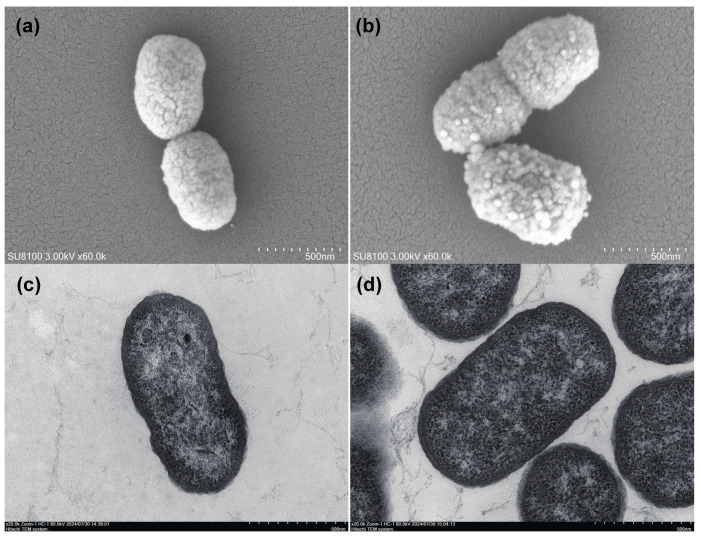
Microscopic characterization of FCF15 and FCF147. (**a**) Morphology of a single FCF15 cell observed under SEM. (**b**) Morphology of a single FCF147 cell observed under SEM. (**c**) Ultrastructure of a single FCF15 cell observed under TEM. (**d**) Ultrastructure of a single FCF147 cell observed under TEM. Detailed imaging conditions are provided in the annotations within each panel.

To conduct a more detailed comparison of the morphological differences between the capsule-deficient strain FCF147 and the capsular type A strain FCF15, we utilized SEM and TEM analyses. SEM imaging indicated that both FCF15 and FCF147 exhibited similar dimensions and morphologies, measuring approximately 500 nm in length and 200 nm in width and presenting as oval-shaped bacilli. Notably, FCF15 demonstrated a smooth and intact surface (Figure 2a), whereas FCF147 was characterized by numerous protrusions and irregular protein-like structures on its surface (Figure 2b), likely attributable to the absence of a protective capsule layer. TEM analysis revealed no discernible differences in cytoplasmic organization or cell membrane thickness between FCF15 and FCF147 (Figure 2c,d), implying that the absence of the capsule predominantly influences surface composition rather than the overall cellular architecture.

### 3.3. Enhancing Biofilm Formation and Tolerance to High Temperature

**Figure 3:**
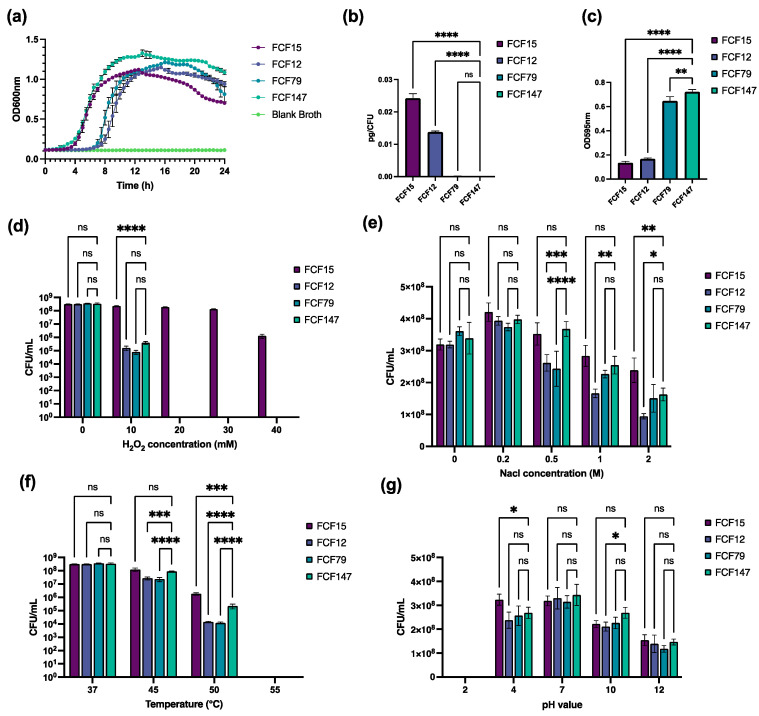
Comparative analysis of in vitro growth dynamics and stress adaptability between FCF147 and representative *P. multocida* strains of distinct genotypes. (**a**) One-step in vitro growth curves. (**b**) Quantitative assessment of CPS. (**c**) Biofilm formation capacity. (**d**) Survival under oxidative stress. (**e**) Survival under hyperosmotic stress. (**f**) Survival under heat stress. (**g**) Survival under acid and alkaline stress. Values are expressed as the mean ± SD, n = 3. *p*-value of <0.05 (*), <0.01 (**), <0.001 (***), or <0.0001 (****). ns: not significant.

To evaluate the in vitro growth and environmental adaptability of FCF147, we conducted a series of phenotypic assays. A one-step in vitro growth curve analysis revealed that FCF147 entered the exponential growth phase earlier than FCF12 and FCF79, although its final cell density in the stationary phase was comparable to that of the other strains (Figure 3a). Capsule quantification assays revealed that FCF147 produced significantly less capsule material (~10^−5^ pg/CFU) compared to FCF12 (capsular type D, ~0.015 pg/CFU) and FCF15 (capsular type A, ~0.025 pg/CFU). Its capsule production was also 10-fold lower than that of FCF79 (capsular type F, ~10^−4^ pg/CFU) (Figure 3b). Conversely, FCF147 demonstrated significantly enhanced biofilm formation relative to other *P. multocida* strains (Figure 3c).

Under conditions of oxidative stress (0/10/20/30/40 mM H_2_O_2_), FCF147 exhibited similar survival rates comparable to those of FCF12 and FCF79. Notably, the dominant A: L1: ST129 strain FCF15 displayed remarkable tolerance to elevated concentrations of H_2_O_2_ (Figure 3d). In contrast, FCF147 showed no significant differences in survival rates under hyperosmotic, acidic, or alkaline stress conditions compared to other strains (Figure 3e,g). However, at higher temperatures (45 °C and 50 °C), FCF147 exhibited significantly greater heat tolerance than other *P. multocida* strains, maintaining high survival rates under these conditions (Figure 3f). Furthermore, MIC testing confirmed that FCF147 remained susceptible to all 14 commonly used veterinary antibiotics (Table 1).

### 3.4. Attenuated Virulence and Limited Immunoprotection in Animal Models

**Figure 4:**
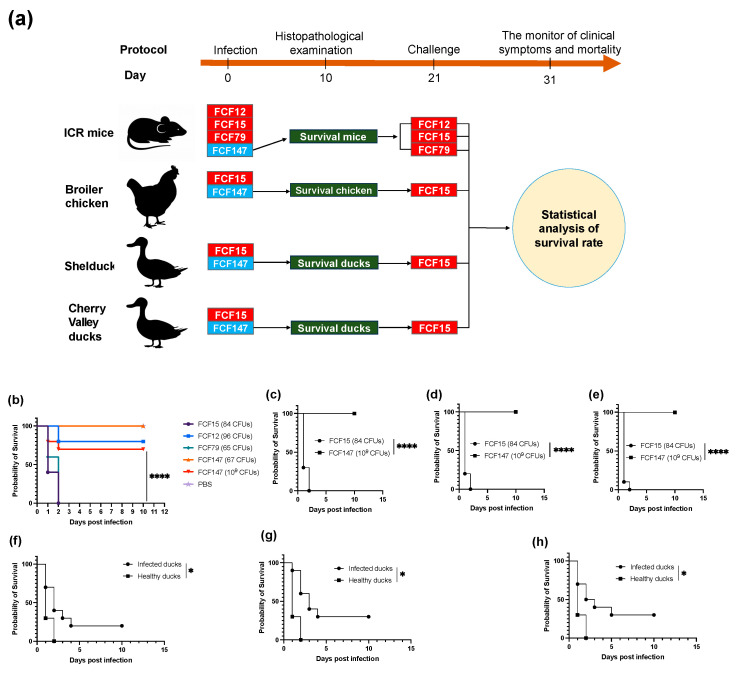
Experimental design and results evaluating the pathogenicity and immunoprotective potential of FCF147. (**a**) Schematic overview of the pathogenicity and immune protection assay design. (**b**) Survival curves of mice challenged with FCF147 and representative *P. multocida* strains of various serotypes. (**c**–**e**) Survival curves of yellow-feather broilers, shelducks, and Cherry Valley ducks challenged with FCF147 and FCF15. (**f**–**h**) Survival curves of yellow-feather broilers, shelducks, and Cherry Valley ducks re-challenged with FCF15 following prior high-dose exposure to FCF147. Statistical significance: *p* < 0.05 (*), *p* < 0.0001 (****).

To evaluate the pathogenicity of the capsule-deficient *P. multocida* strain FCF147, infection experiments were conducted in mice (Figure 4a). The results indicated that all mice survived five days post-infection with a low dose of FCF147 (67 CFU), and even with a high dose (~10^9^ CFU), the survival rate remained as high as 70%. In contrast, FCF15 and FCF79 exhibited significantly higher lethality, underscoring the attenuated virulence of FCF147 (Figure 4b). Additionally, the virulence of FCF147 was compared to FCF15, the predominant *P. multocida* clone in Chinese poultry (Figure 4a). Rapid mortality was observed in yellow-feather broilers (Figure 4c), shelducks (Figure 4d), and Cherry Valley ducks (Figure 4e) following intramuscular inoculation with FCF15 (84 CFU). However, all poultry inoculated with a high dose of FCF147 (~10^9^ CFU) survived for at least ten days, suggesting that FCF147 has lost its ability to cause disease in poultry.

To evaluate the potential of FCF147 exposure to elicit protective immunity, we conducted bacterial challenge experiments on surviving mice and poultry previously inoculated with FCF147 at approximately 10^9^ CFU (Figure 4a). The findings indicated that mice surviving FCF147 exposure rapidly succumbed when challenged with low doses of FCF12 (83 CFU), FCF15 (66 CFU), or FCF79 (72 CFU) (Figure S4a–c). Similarly, poultry that survived FCF147 exposure demonstrated limited protection against an FCF15 challenge (75 CFU), with 10-day survival rates of only 20% in yellow-feather broilers, 30% in shelducks, and 30% in Cherry Valley ducks.

**Figure 5:**
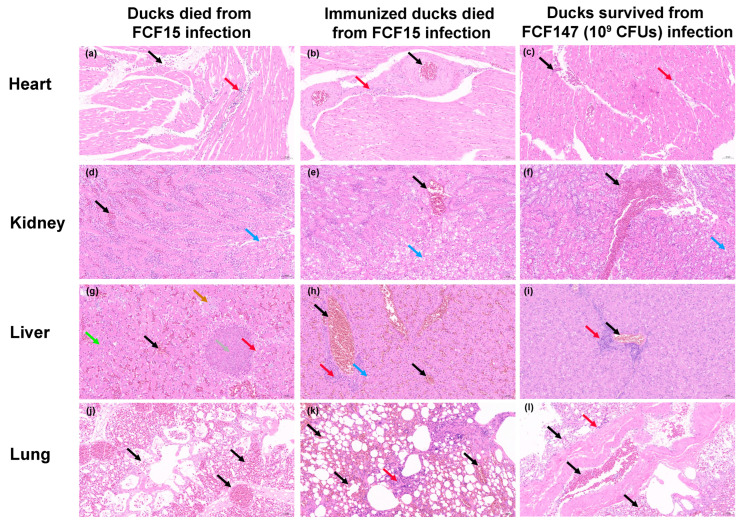
Histopathological analysis of tissues from ducks subjected to pathogenicity and immune protection assays (H&E staining; scale bar = 50 μm). Black arrows indicate vascular congestion and erythrocyte infiltration; red arrows indicate lymphocyte infiltration; blue arrows indicate cellular hydropic degeneration; green arrows indicate fatty degeneration; brown arrows denote basophilic aggregates; and grey arrows indicate focal necrosis. (**a**–**c**) Heart tissues. (**d**–**f**) Kidney tissues. (**g**–**i**) Liver tissues. (**j**–**l**) Lung tissues.

To evaluate the pathogenicity and immunoprotective efficacy of FCF147 in poultry, histopathological analyses were performed on three groups of ducks: those that succumbed to FCF15 infection, survivors of FCF147 infection, and FCF147-immunized ducks that later died following FCF15 challenge. Myocardial architecture remained largely intact across all groups; however, pronounced infiltration of erythrocytes and lymphocytes was observed in the hearts of FCF15-infected ducks, whereas only mild infiltration was noted in FCF147 survivors (Figure 5a–c). Renal lesions were mild and comparable among the groups, characterized by slight hydropic degeneration and limited inflammatory infiltration (Figure 5d–f). Liver pathology in FCF15-infected ducks revealed severe sinusoidal and venous congestion, hepatocellular necrosis, and fatty degeneration (Figure 5g). In contrast, FCF147 survivors and immunized ducks exhibited only mild hepatocellular alterations, with reduced inflammation and minimal fatty changes (Figure 5h,i). Pulmonary lesions varied among groups. FCF15-infected ducks showed severe vascular congestion with minimal inflammatory infiltration, indicative of acute lung injury (Figure 5j). Conversely, lungs from FCF147 survivors and immunized ducks displayed prominent inflammatory cell infiltration, reflecting an active immune response (Figure 5k,l). Notably, immunized ducks that later succumbed to FCF15 exhibited marked alveolar enlargement and septal thickening, suggestive of compensatory emphysema and chronic inflammatory repair.

These findings confirm that FCF147 exhibits significantly attenuated virulence in poultry. Although prior exposure to FCF147 elicits a partial immune response, it fails to confer effective protection against subsequent FCF15 infection, as evidenced by continued susceptibility and limited histopathological improvement.

## 4. Discussion

In this study, we identified and characterized *P. multocida* strain FCF147, which was isolated from a black-necked swan mortality event (Figure S1). Although most studies on *P. multocida* have focused on the epidemiology and transmission in livestock, its potential threat to wildlife, particularly endangered species, should not be overlooked. For instance, *P. multocida* strains carrying a range of virulence factors associated with human and animal diseases have been implicated in mass mortality events in African elephants [6]. Similarly, outbreaks of *P. multocida* capsular serogroup B strains have led to the deaths of over 200,000 saiga antelopes (*Saiga tatarica*) in Kazakhstan [5,36]. The growing number of human infections attributed to *P. multocida* has further raised awareness of its underrecognized public health risks [2].

The capsular serotype, LPS genotype, and MLST of *P. multocida* are known to correlate strongly with its geographic distribution and host preference [4]. In our previous work, we identified the predominant poultry-associated clone A:L1:ST129 in China [18]. Phylogenetic analysis showed that FCF147 is genetically distant from most known *P. multocida* strains and does not cluster within any major phylogenetic lineage, including the dominant poultry-associated A:L1:ST129 clone in China (Figure 1a). Although FCF147 was classified as an L2-type LPS strain, it could not be assigned to any known capsular serogroup or MLST type, underscoring its genomic distinctiveness. This genomic divergence is further supported by the enrichment of genes involved in cell motility, primary metabolism, and secondary metabolite biosynthesis, which may contribute to the strain’s adaptation and survival in specific environmental hosts [1,4]. One of the most striking genomic features of FCF147 is the complete absence of the capsular biosynthesis locus (Figure 1b). While capsule-deficient or untypeable *P. multocida* strains have previously been reported in isolates from pets or humans [23], this is the first such report in a strain isolated from avian hosts, especially from wildlife. Notably, FCF147 is similar to the strains described by Thomas R. Smallman et al. [23], as both were isolated from natural environments, exhibited complete loss of the capsule biosynthesis locus, and belonged to L2/L3-type LPS backgrounds. In contrast, another study reported capsule-deficient strains derived from capsular type A backgrounds, in which serial in vitro passage led to fis gene mutations that downregulated capsule gene expression [24]. Based on these comparisons and consistent with Smallman et al.’s conclusions, we speculate that the capsule may not be strictly required for *P. multocida* to cause certain diseases in animals or humans. The deletion of the entire capsule biosynthesis cluster, including genes involved in phospholipid substitution and export, suggests that FCF147 has undergone different evolutionary pressures to adapt to its ecological niche.

The capsule plays a pivotal role in the pathogenicity of *P. multocida* by facilitating immune evasion, enhancing resistance to phagocytosis, and promoting environmental persistence [17]. The complete loss of the capsule gene cluster in FCF147 has profound implications for its surface morphology. Unlike the smooth surface of the capsular type A strain FCF15, FCF147 exhibits numerous protrusions and irregular, protein-like structures. This suggests that in the absence of a protective capsule, surface-associated proteins and adhesins may be exposed, potentially altering host-pathogen interactions and environmental adaptability. The exposure of bacterial surface proteins can promote binding to host-specific fibrinogen, thereby facilitating biofilm formation and evasion of innate immune responses [37]. A well-characterized example of this is the RTX adhesin, which enhances biofilm development and contributes to bacterial persistence [38]. This mechanism may help explain, at least in part, the significantly enhanced biofilm-forming capacity and reduced pathogenicity of FCF147.

One of the most striking phenotypic differences observed in FCF147 compared to other *P. multocida* strains is its significantly enhanced biofilm-forming ability. Biofilm formation is a key virulence and survival strategy in many bacterial pathogens, enabling resistance to environmental stresses and antimicrobial agents [34,39]. This observation is consistent with previous findings that capsule-deficient *P. multocida* strains can exhibit enhanced biofilm production [34,39], which may contribute to prolonged environmental persistence and colonization of new hosts. However, the specific mechanisms by which capsule reduction or loss enhances biofilm formation remain to be elucidated in future studies. Furthermore, another study demonstrated that biofilms formed by *P. multocida* induced only mild inflammatory responses in organs such as the lungs, liver, and heart, supporting the development of chronic fowl cholera infections in avian species [40]. This aligns with our histopathological observations, where ducks infected with FCF147 displayed milder pathological changes compared to those infected with the virulent FCF15 strain (Figure 5). In addition, capsule loss may influence bacterial interactions with host cells. A previous study showed that capsule-deficient *P. multocida* mutants exhibited altered host–cell interaction patterns, potentially due to changes in surface properties affecting adhesion capabilities [41]. Capsule deficiency may also be linked to iron acquisition mechanisms, as iron is essential for bacterial growth and replication. The absence of a capsule could affect iron uptake and utilization, thereby influencing bacterial survival within the host [42].

The ability of *P. multocida* to survive under various environmental stresses is critical for its transmission and pathogenicity. Our results show that FCF147 exhibits environmental tolerance similar to other *P. multocida* strains but with notably enhanced resistance to heat stress. This increased thermotolerance suggests that FCF147 may possess alternative mechanisms for protein stabilization and membrane integrity maintenance at elevated temperatures, potentially contributing to its survival in warmer climates or in avian hosts with higher basal body temperatures. Such stress-resilient phenotypes may be linked to global regulators such as two-component systems. For example, deletion of the histidine kinase gene *qseC* in the QseBC quorum sensing system has been reported to significantly reduce capsule production and virulence in *P. multocida* while simultaneously enhancing resistance to oxidative and osmotic stresses [43].

Despite its enhanced biofilm formation and thermotolerance, FCF147 does not harbor any known antimicrobial resistance genes and remains susceptible to all 14 commonly used antibiotics (Table 1). This is a critical finding, as antimicrobial resistance in *P. multocida* is an increasing concern in both livestock and wildlife management. The absence of resistance determinants in FCF147 suggests that it has neither acquired nor maintained the genetic elements conferring antibiotic resistance, further distinguishing it from epidemic *P. multocida* strains typically associated with livestock infections. Antimicrobial resistance is often complex and severe among *P. multocida* isolates from economic animals [4,18,44] and is considered a driving factor in the emergence of multidrug-resistant strains in production systems. This finding supports the hypothesis that the wild swan-derived FCF147 has not been subjected to strong antibiotic selection pressures.

Compared to other *P. multocida* strains, particularly the highly virulent FCF15, FCF147 exhibits significantly attenuated virulence (Figure 4). In murine infection models, even at high inoculum doses (~10^9^ CFU), FCF147 induced minimal mortality, whereas FCF15 and other strains caused markedly higher lethality (Figure 5). Similarly, in avian infection models, FCF147 failed to induce disease in yellow-feather broilers, shelducks, or Cherry Valley ducks, even at high-challenge doses. The lack of a capsule is likely one of the major factors contributing to its drastically reduced pathogenicity in poultry. Furthermore, the L2-type LPS found in FCF147 is typically associated with hemorrhagic septicaemia in cattle [45], which may also contribute to its limited virulence in both avian and murine hosts.

Histopathological analysis further confirmed the attenuated virulence of FCF147 (Figure 5). Ducks that survived FCF147 infection showed minimal tissue damage and only mild inflammatory responses, in stark contrast to the severe lesions observed in ducks that succumbed to FCF15 infection. This suggests that, although FCF147 is capable of colonizing avian hosts, it does not elicit the severe inflammatory responses typically associated with highly virulent *P. multocida* strains. Notably, while FCF147 exposure triggered an immune response, it failed to confer effective protection against subsequent lethal challenges with FCF15. The protection rate offered by FCF147 immunization in poultry was only around 30%, compared to up to 50% achieved by a serially passaged attenuated type A *P. multocida* vaccine strain constructed by He et al. [46]. This suggests that the loss of the capsule in FCF147 may also impair its immunogenicity, limiting its potential as a naturally attenuated vaccine candidate. Several factors may contribute to the failure of immunoprotection. For instance, *P*. *multocida* toxin (PMT) has been shown to disrupt the differentiation and function of immune cells, thereby impairing the host’s immune response [47]. In addition, the choice of immunization route may also influence the effectiveness of immune protection. Studies have demonstrated that mucosal immunization against *P. multocida* provides better protective efficacy compared to subcutaneous immunization [48].

The findings of this study may be generalizable to other avian species infected by *P. multocida*, particularly within environments where capsule-deficient strains can persist and adapt. While the FCF147 strain was isolated from a swan, its attenuated virulence observed in chickens and ducks suggests broader ecological and epidemiological relevance across poultry. However, direct extrapolation to mammalian hosts or human infections is limited due to species-specific immune responses and differences in host–pathogen interactions. Further studies are needed to evaluate the behavior of similar capsule-deficient strains in different host species and environmental contexts. This study has several limitations. First, the sample size, although based on ethical and practical considerations, may limit the statistical power for detecting subtle phenotypic differences. Second, while mice and poultry are commonly used models for *P. multocida* infection, they may not fully replicate the pathogenesis in wild avian hosts, especially swans. Third, despite efforts to control variables such as housing and environmental conditions, unrecognized confounders (e.g., microbiota variation or animal handling stress) may have influenced the results. Finally, while in vitro assays were informative, additional in vivo experiments (e.g., long-term colonization or transmission studies) would further clarify the ecological impact of FCF147.

## 5. Conclusions

The isolation of a capsule-deficient *P. multocida* strain from a black-necked swan highlights the potential emergence of unique *P. multocida* variants within wild animal populations. The genetic and phenotypic characteristics of FCF147 suggest that it may represent an evolutionary branch of *P. multocida* that favors environmental persistence over systemic virulence. Its enhanced biofilm formation and thermotolerance may promote survival in wetlands and warmer habitats, enabling it to persist in reservoirs that could sporadically trigger outbreaks among wild birds and possibly contribute to chronic infections in poultry through environmental transmission. Understanding the ecological roles of such attenuated *P. multocida* strains is crucial for wildlife conservation efforts and epidemiological surveillance. The presence of capsule-deficient variants in wild bird populations may indicate an underappreciated reservoir of low-virulence *P. multocida* strains, which could potentially recombine with more virulent counterparts and contribute to the emergence of novel pathogens.

## Figures and Tables

**Table 1:** MIC values of FCF12/FCF15/FCF79/FCF147 against 14 antimicrobial agents.

Antimicrobial Agents	FCF12	FCF15	FCF79	FCF147
Ampicillin	<0.25	<0.25	0.5	<0.25
Amoxicillin	0.5	0.5	0.5	0.5
Kanamycin	0.5	8	4	1
Streptomycin	1	4	1	1
Erythromycin	1	2	2	2
Tilmicosin	2	4	2	1
Chloramphenicol	0.5	1	1	0.5
Florfenicol	1	0.5	1	0.5
Tetracycline	1	16	4	2
Tigecycline	<0.25	<0.25	<0.25	<0.25
Sulfadiazine	2	>128	4	4
Ciprofloxacin	0.5	1	1	1
Enrofloxacin	<0.25	0.5	1	0.5
Rifampin	<0.25	<0.25	<0.25	<0.25

## Data Availability

The datasets generated and/or analyzed during the current study are available from the corresponding author upon reasonable request. WGS data of strain FCF147 have been deposited in the NCBI under accession number NZ_CP143490.1. All gene sequences in this study are available in the NCBI database using the accession numbers in Table S1.

## Supplementary Materials

The following supporting information can be downloaded at https://www.mdpi.com/article/10.3390/microorganisms13051024/s1, Figure S1: Observation of swan carcasses and lesions; Figure S2: Completed genome map of FCF147; Figure S3: FCF147 genome annotation; Figure S4: Immunoprotective test of FCF147 in mice; Table S1: Detailed information for all genomes in this study.

## Abbreviations

The following abbreviations are used in this manuscript:

LPS	Lipopolysaccharide
MLST	The multi-locus sequence typing
TSA	Tryptic soy agar
TSB	Tryptic soy broth
FBS	Fetal bovine serum
WGS	Whole genome sequencing
GO	The Gene Ontology
KEGG	The Kyoto Encyclopedia of Genes and Genomes
CARD	The Comprehensive Antibiotic Resistance Database
VFDB	The Virulence Factor Database
PHI	The Pathogen Host Interactions
ML	Maximum likelihood
GTR	General time-reversible
TEM	Transmission electron microscope
SEM	Scanning electron microscope
PBS	Phosphate-buffered saline
OD	The optical density
rpm	Revolutions per minute
CFU	colony-forming unit
CV	Crystal Violet
MIC	Minimum Inhibitory Concentration
CA-MHB	Cation-adjusted Mueller–Hinton broth
HE	Hematoxylin and eosin
SDs	Standard deviations
ns	Not significant

## References

[1] Hurtado R., Maturrano L., Azevedo V., Aburjaile F. (2020). Pathogenomics insights for understanding *Pasteurella multocida* adaptation. Int. J. Med. Microbiol..

[2] Peng Z., Lin L., Wang X., Chen H., Wu B. (2022). The public health concern of *Pasteurella multocida* should not be ignored. Lancet Microbe.

[3] Wilson B.A., Ho M. (2013). *Pasteurella multocida*: From zoonosis to cellular microbiology. Clin. Microbiol. Rev..

[4] Peng Z., Wang X., Zhou R., Chen H., Wilson B.A., Wu B. (2019). *Pasteurella multocida*: Genotypes and Genomics. Microbiol. Mol. Biol. Rev..

[5] Fereidouni S., Freimanis G.L., Orynbayev M., Ribeca P., Flannery J., King D.P., Zuther S., Beer M., Hoper D., Kydyrmanov A. (2019). Mass Die-Off of Saiga Antelopes, Kazakhstan, 2015. Emerg. Infect. Dis..

[6] Foggin C.M., Rosen L.E., Henton M.M., Buys A., Floyd T., Turner A.D., Tarbin J., Lloyd A.S., Chaitezvi C., Ellis R.J. (2023). *Pasteurella sp*. associated with fatal septicaemia in six African elephants. Nat. Commun..

[7] Hashish A., Johnson T.J., Chundru D., Williams M.L., Sato Y., Macedo N.R., Clessin A., Gantelet H., Bost C., Tornos J. (2023). Complete Genome Sequences of Two *Pasteurella multocida* Isolates from Seabirds. Microbiol. Resour. Announc..

[8] Smith O.M., Snyder W.E., Owen J.P. (2020). Are we overestimating risk of enteric pathogen spillover from wild birds to humans?. Biol. Rev. Camb. Philos. Soc..

[9] Plowright R.K., Parrish C.R., McCallum H., Hudson P.J., Ko A.I., Graham A.L., Lloyd-Smith J.O. (2017). Pathways to zoonotic spillover. Nat. Rev. Microbiol..

[10] Cohen J. (2024). Virus gone wild. Science.

[11] Hubalek Z. (2004). An annotated checklist of pathogenic microorganisms associated with migratory birds. J. Wildl. Dis..

[12] Samuel M.D., Goldberg D.R., Shadduck D.J., Price J.I., Cooch E.G. (1997). *Pasteurella multocida* serotype 1 isolated from a lesser snow goose: Evidence of a carrier state. J. Wildl. Dis..

[13] Samuel M.D., Shadduck D.J., Goldberg D.R. (2005). Avian cholera exposure and carriers in greater white-fronted geese breeding in Alaska, USA. J. Wildl. Dis..

[14] Samuel M.D., Shadduck D.J., Goldberg D.R., Johnson W.P. (2005). Avian cholera in waterfowl: The role of lesser snow and ross’s geese as disease carriers in the Playa Lakes Region. J. Wildl. Dis..

[15] Pritchett I.W., Hughes T.P. (1932). The Epidemiology of Fowl Cholera: Vi. The Spread of Epidemic and Endemic Strains of *Pasteurella Avicida* in Laboratory Populations of Normal Fowl. J. Exp. Med..

[16] Samuel M.D., Shadduck D.J., Goldberg D.R., Johnson W.P. (2003). Comparison of methods to detect *Pasteurella multocida* in carrier waterfowl. J. Wildl. Dis..

[17] Guan L., Zhang L., Xue Y., Yang J., Zhao Z. (2020). Molecular pathogenesis of the hyaluronic acid capsule of *Pasteurella multocida*. Microb. Pathog..

[18] Jiang N., Chen H., Cheng L., Fu Q., Liu R., Liang Q., Fu G., Wan C., Huang Y. (2024). Genomic analysis reveals the population structure and antimicrobial resistance of avian *Pasteurella multocida* in China. J. Antimicrob. Chemother..

[19] Boyce J.D., Adler B. (2000). The capsule is a virulence determinant in the pathogenesis of *Pasteurella multocida* M1404 (B:2). Infect. Immun..

[20] Chung J.Y., Wilkie I., Boyce J.D., Townsend K.M., Frost A.J., Ghoddusi M., Adler B. (2001). Role of capsule in the pathogenesis of fowl cholera caused by *Pasteurella multocida* serogroup A. Infect. Immun..

[21] Li N., Feng T., Wang Y., Li P., Yin Y., Zhao Z., Hardwidge P.R., Peng Y., He F. (2021). A single point mutation in the *hyaC* gene affects *Pasteurella multocida* serovar A capsule production and virulence. Microb. Pathog..

[22] Tang X., Zhao Z., Hu J., Wu B., Cai X., He Q., Chen H. (2009). Isolation, antimicrobial resistance, and virulence genes of *Pasteurella multocida* strains from swine in China. J. Clin. Microbiol..

[23] Smallman T.R., Perlaza-Jimenez L., Wang X., Korman T.M., Kotsanas D., Gibson J.S., Turni C., Harper M., Boyce J.D. (2024). Pathogenomic analysis and characterization of *Pasteurella multocida* strains recovered from human infections. Microbiol. Spectr..

[24] Steen J.A., Steen J.A., Harrison P., Seemann T., Wilkie I., Harper M., Adler B., Boyce J.D. (2010). Fis is essential for capsule production in *Pasteurella multocida* and regulates expression of other important virulence factors. PLoS Pathog..

[25] Thomas P.D., Ebert D., Muruganujan A., Mushayahama T., Albou L.P., Mi H. (2022). PANTHER: Making genome-scale phylogenetics accessible to all. Protein Sci..

[26] Kanehisa M., Furumichi M., Sato Y., Matsuura Y., Ishiguro-Watanabe M. (2025). KEGG: Biological systems database as a model of the real world. Nucleic Acids Res..

[27] Alcock B.P., Huynh W., Chalil R., Smith K.W., Raphenya A.R., Wlodarski M.A., Edalatmand A., Petkau A., Syed S.A., Tsang K.K. (2023). CARD 2023: Expanded curation, support for machine learning, and resistome prediction at the Comprehensive Antibiotic Resistance Database. Nucleic Acids Res..

[28] Zhou S., Liu B., Zheng D., Chen L., Yang J. (2025). VFDB 2025: An integrated resource for exploring anti-virulence compounds. Nucleic Acids Res..

[29] Cuzick A., Seager J., Wood V., Urban M., Rutherford K., Hammond-Kosack K.E. (2023). A framework for community curation of interspecies interactions literature. eLife.

[30] Carattoli A., Zankari E., Garcia-Fernandez A., Voldby Larsen M., Lund O., Villa L., Moller Aarestrup F., Hasman H. (2014). In silico detection and typing of plasmids using PlasmidFinder and plasmid multilocus sequence typing. Antimicrob. Agents Chemother..

[31] Camacho C., Coulouris G., Avagyan V., Ma N., Papadopoulos J., Bealer K., Madden T.L. (2009). BLAST+: Architecture and applications. BMC Bioinform..

[32] Stamatakis A. (2014). RAxML version 8: A tool for phylogenetic analysis and post-analysis of large phylogenies. Bioinformatics.

[33] Jiang N., Wyres K.L., Li J., Fessler A.T., Kruger H., Wang Y., Holt K.E., Schwarz S., Wu C. (2021). Evolution and genomic insight into methicillin-resistant *Staphylococcus aureus* ST9 in China. J. Antimicrob. Chemother..

[34] Lee Y.J., Cao D., Subhadra B., De Castro C., Speciale I., Inzana T.J. (2024). Relationship between capsule production and biofilm formation by *Mannheimia haemolytica*, and establishment of a poly-species biofilm with other Pasteurellaceae. Biofilm.

[35] Clinical and Laboratory Standards Institute (2024). Performance Standards for Antimicrobial Susceptibility Testing.

[36] Kock R.A., Orynbayev M., Robinson S., Zuther S., Singh N.J., Beauvais W., Morgan E.R., Kerimbayev A., Khomenko S., Martineau H.M. (2018). Saigas on the brink: Multidisciplinary analysis of the factors influencing mass mortality events. Sci. Adv..

[37] Pickering A.C., Vitry P., Prystopiuk V., Garcia B., Höök M., Schoenebeck J., Geoghegan J.A., Dufrêne Y.F., Fitzgerald J.R. (2019). Host-specialized fibrinogen-binding by a bacterial surface protein promotes biofilm formation and innate immune evasion. PLoS Pathog..

[38] Guo S., Vance T.D.R., Stevens C.A., Voets I.K., Davies P.L. (2019). RTX Adhesins are Key Bacterial Surface Megaproteins in the Formation of Biofilms. Trends Microbiol..

[39] Petruzzi B., Briggs R.E., Tatum F.M., Swords W.E., De Castro C., Molinaro A., Inzana T.J. (2017). Capsular Polysaccharide Interferes with Biofilm Formation by *Pasteurella multocida* Serogroup A. mBio.

[40] Petruzzi B., Dalloul R.A., LeRoith T., Evans N.P., Pierson F.W., Inzana T.J. (2018). Biofilm formation and avian immune response following experimental acute and chronic avian cholera due to *Pasteurella multocida*. Vet. Microbiol..

[41] Su A., Tong J., Fu Y., Muller S., Weldearegay Y.B., Becher P., Valentin-Weigand P., Meens J., Herrler G. (2020). Infection of bovine well-differentiated airway epithelial cells by *Pasteurella multocida*: Actions and counteractions in the bacteria-host interactions. Vet. Res..

[42] Shen X., Guan L., Zhang J., Xue Y., Si L., Zhao Z. (2025). Study in the iron uptake mechanism of *Pasteurella multocida*. Vet. Res..

[43] Yang Y., Hu P., Gao L., Yuan X., Hardwidge P.R., Li T., Li P., He F., Peng Y., Li N. (2021). Deleting qseC downregulates virulence and promotes cross-protection in *Pasteurella multocida*. Vet. Res..

[44] Shi C., Zhu Z., Shang Y., Song W., Yang J., Bi H., Wang Z., Xie R., Zhao M., Hua L. (2024). Discovery of the tigecycline resistance gene cluster tmexCD3-toprJ1 in *Pasteurella multocida* strains isolated from pigs in China. Vet. Microbiol..

[45] Moustafa A.M., Seemann T., Gladman S., Adler B., Harper M., Boyce J.D., Bennett M.D. (2015). Comparative Genomic Analysis of Asian Haemorrhagic Septicaemia-Associated Strains of *Pasteurella multocida* Identifies More than 90 Haemorrhagic Septicaemia-Specific Genes. PLoS ONE.

[46] He F., Xiong P., Zhang H., Yang L., Qiu Y., Li P., Zhao G., Li N., Peng Y. (2024). Attenuated vaccine PmCQ2Delta4555-4580 effectively protects mice against *Pasteurella multocida* infection. BMC Vet. Res..

[47] Kubatzky K.F., Kloos B., Hildebrand D. (2013). Signaling cascades of *Pasteurella multocida* toxin in immune evasion. Toxins.

[48] Kharb S., Charan S. (2011). Mucosal immunization provides better protection than subcutaneous immunization against *Pasteurella multocida* (B:2) in mice preimmunized with the outer membrane proteins. Vet. Res. Commun..

