# Supplementary material for: Characterization of a Capsule-Deficient Pasteurella multocida Isolated from Cygnus melancoryphus: Genomic, Phenotypic, and Virulence Insights
Source: Microorganisms. 2025 Apr 29;13(5):1024. doi: 10.3390/microorganisms13051024 (PMC12113756; doi:10.3390/microorganisms13051024)
Supplement: Supplementary file 1 [file microorganisms-13-01024-s001.zip › microorganisms-3600597-supplementary.pdf]

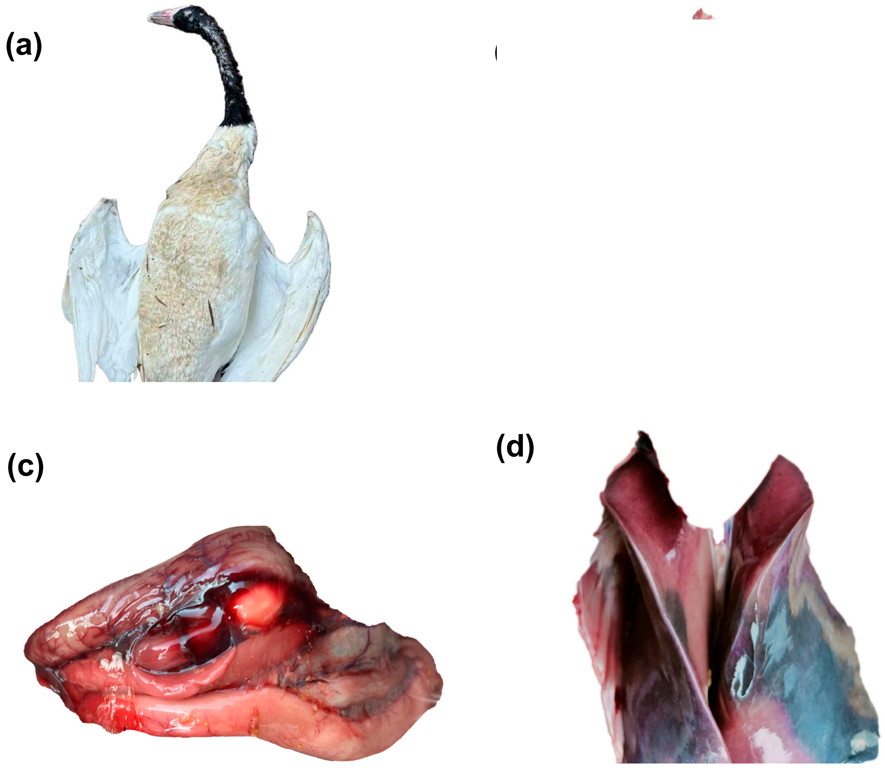

Figure S1. Observation of swan carcasses and lesions (a) Black-necked swans that succumbed to avian cholera; (b) Pericardial hemorrhage with numerous hemorrhagic spots on the epicardium and coronary fat; (c) Intestinal swelling with extensive mucosal hemorrhage and gelatinous intestinal contents; (d) Severe hepatic necrosis.

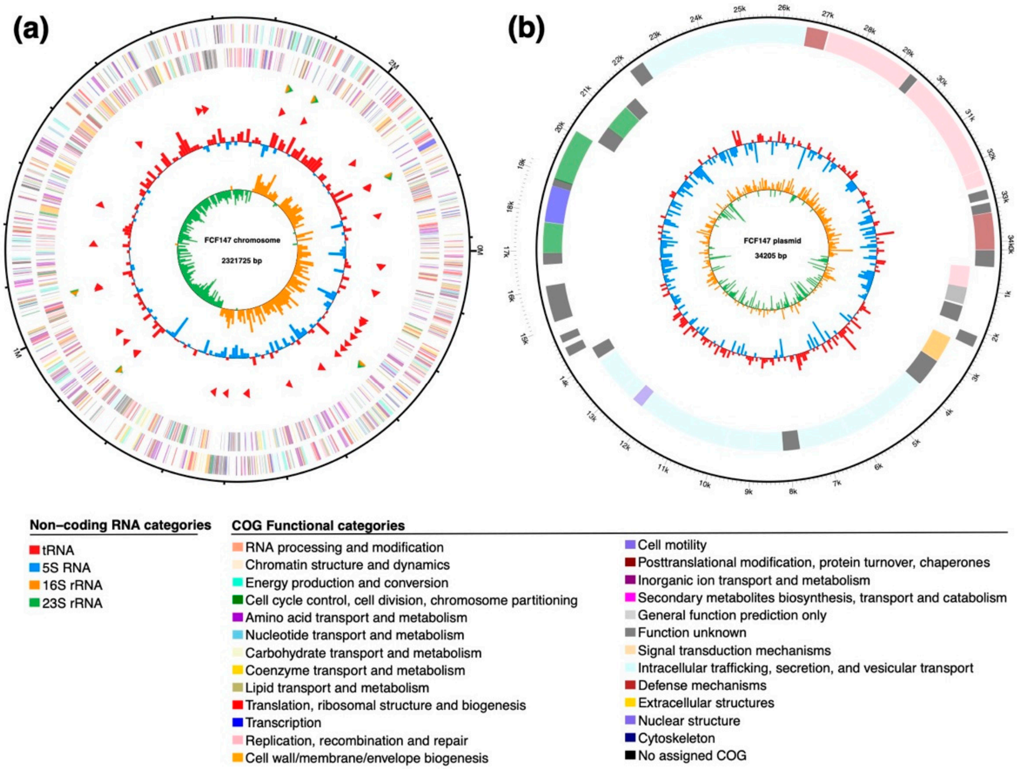

Figure S2. Completed genome map of FCF147. (a) Chromosomal genome sequence; (b) Plasmid sequence. The outermost ring indicates genome size markers. The second and third rings represent CDSs on the forward and reverse strands, respectively, with colors indicating different COG functional classifications. The fourth ring shows rRNA and tRNA. The fifth ring represents GC content, where red regions extending outward indicate GC content higher than the genome average (with peak height reflecting the degree of deviation), and blue regions extending inward indicate GC content lower than the genome average. The innermost ring displays the GC skew values.

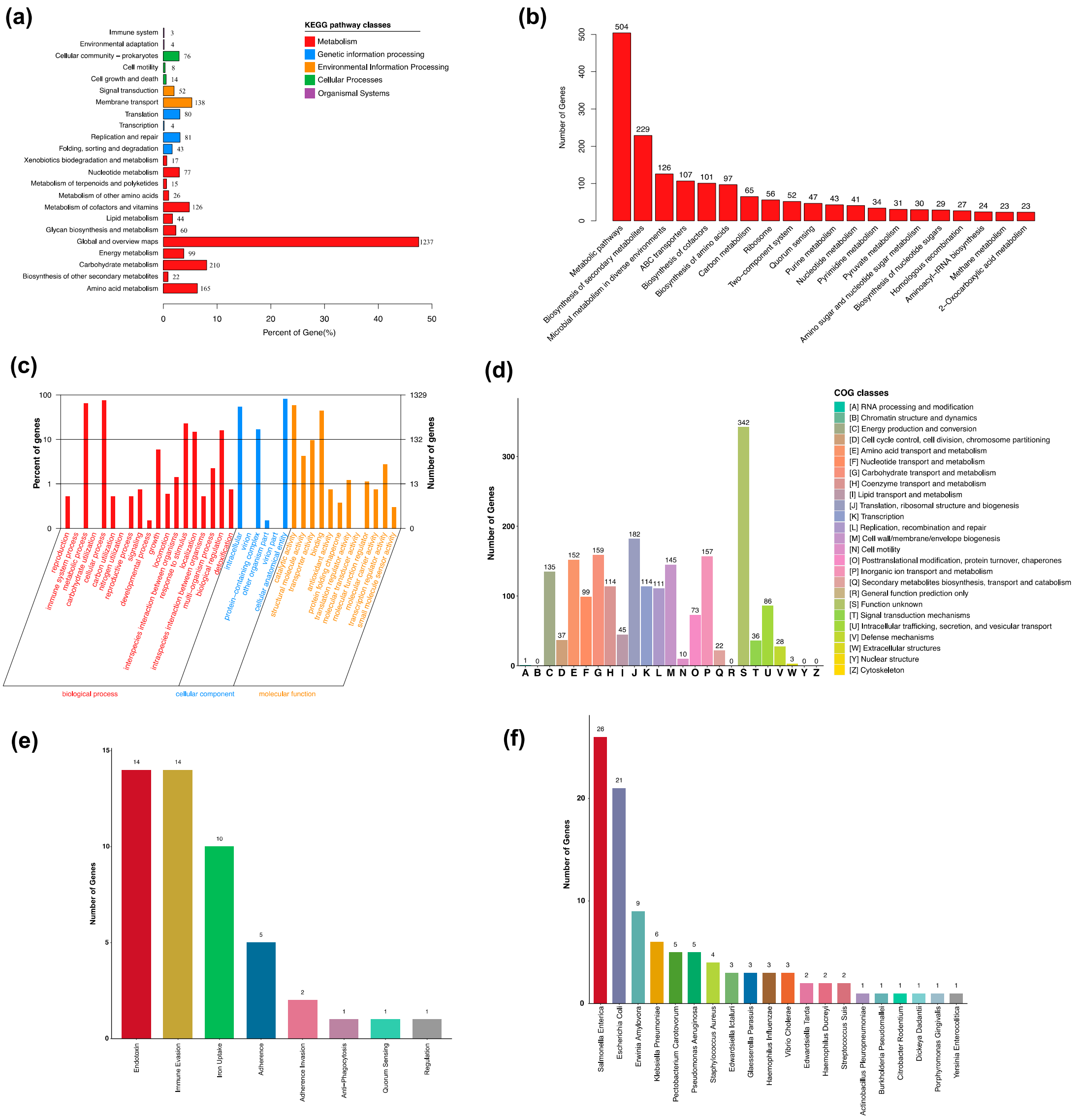

Figure S3. Annotation of FCF147 genes. (a) Enrichment of FCF147 genes in primary and secondary pathways based on KEGG database annotations. (b) Enrichment of FCF147 genes in tertiary pathways based on KEGG database annotations. (c) Functional distribution of FCF147 genes based on GO database annotations. (d) Functional classification of FCF147 genes based on COG database annotations. (e) Classification and number of virulence genes

carried by FCF147 based on VFDB database annotations. (f) Number of homologous pathogenic genes in FCF147 across different bacterial species based on PHI database annotations.

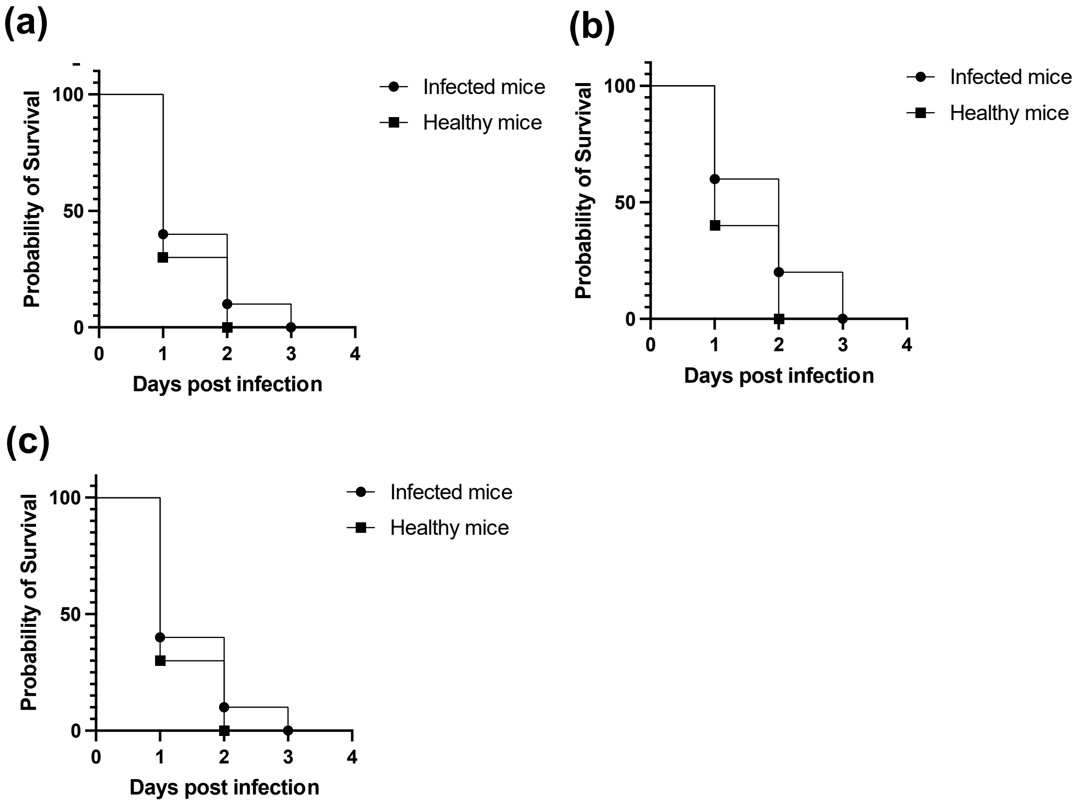

Figure S4. Survival curves of mice in immune protection assay. (a) Survival curves of mice re-challenged with FCF12 following prior high-dose exposure to FCF147. (b) Survival curves of mice re-challenged with FCF15 following prior high-dose exposure to FCF147. (c) Survival curves of mice re-challenged with FCF79 following prior high-dose exposure to FCF147.
